# Coronary CT Angiography for PCI Planning and Guidance: A Comprehensive Narrative Review

**DOI:** 10.3390/medicina62020313

**Published:** 2026-02-03

**Authors:** Lorenzo Fargione, Pietro Laforgia, Thomas Hovasse, Bernard Chevalier, Nicolas Amabile, Francesca Sanguineti, Stephane Champagne, Thierry Unterseeh, Antoinette Neylon, Neila Sayah, Jerome Garot, Lisa Simioni, Mario Togni, Stephane Cook, Hakim Benamer, Livio D’Angelo, Philippe Garot, Mariama Akodad, Ioannis Skalidis

**Affiliations:** 1 Institut Cardiovasculaire Paris Sud (ICPS), Hôpital Privé Jacques Cartier, 91300 Massy, France; 2 Department of Cardiology, HFR Fribourg Cantonal Hospital and University, 1708 Fribourg, Switzerland

**Keywords:** CCTA, PCI planning, CT-guided PCI, coronary artery disease, FFR-CT, virtual PCI, calcium modification, bifurcation lesions, chronic total occlusion, intravascular imaging, non invasive imaging, cardiac imaging, coronary artery disease

## Abstract

Coronary computed tomography angiography (CCTA) is increasingly recognized as a comprehensive tool for planning percutaneous coronary intervention (PCI). By integrating plaque morphology, calcium burden, and CT-derived coronary physiology, CCTA enables non-invasive assessment of lesion complexity and supports precision-guided revascularization. This narrative review synthesizes current evidence on CT-guided PCI from original studies, registries, expert consensus documents, and international guideline recommendations. The literature was identified through PubMed, Embase, and Google Scholar, focusing on CCTA-based plaque characterization, calcium assessment, bifurcation and ostial lesions, chronic total occlusions (CTO), FFR-CT, virtual PCI simulation, and fusion imaging. Particular attention was given to contemporary investigations such as SYNTAX III, P3, and the ongoing P4 trial. CCTA reliably characterizes stenosis severity, plaque distribution, and calcification, demonstrating strong concordance with intravascular imaging. CT-based measurements support accurate stent sizing, prediction of calcium modification requirements, and identification of high-risk features in bifurcation and ostial disease. In CTO PCI, CCTA enhances visualization of proximal cap morphology, occlusion length, tortuosity, and distal vessel quality, outperforming angiographic scoring systems. CT-derived physiology and virtual PCI planning improve lesion selection and allow prediction of post-PCI hemodynamics. Emerging technologies—including photon-counting CT, artificial intelligence-assisted plaque analysis, and CT–fluoroscopy fusion—further expand the applicability of CT-guided PCI. The ongoing P4 trial is expected to provide definitive validation of CT-guided PCI and may support its incorporation into routine clinical workflows.

## 1. Introduction

Coronary CT angiography (CCTA) has demonstrated high diagnostic accuracy for detecting coronary artery disease (CAD) and plays a key role in the management of patients with low-to-intermediate pre-test likelihood of CAD [1]. Over the past decade, technological advances in scanner hardware, image reconstruction, and post-processing software have substantially improved spatial and temporal resolution, enabling the detailed non-invasive assessment of coronary anatomy and plaque characteristics. As a result, an increasing number of patients are referred to the catheterization laboratory following a diagnostic workup based on CCTA, particularly in the setting of chronic coronary syndromes (CCS).

Despite this evolution, percutaneous coronary intervention (PCI) planning continues to rely predominantly on invasive coronary angiography (ICA), which provides limited two-dimensional information and may underestimate lesion complexity, plaque morphology, and vessel geometry. This intrinsic limitation can translate into suboptimal procedural planning, inappropriate device selection, increased contrast use, prolonged procedural times, and a higher risk of procedural complications, particularly in complex scenarios such as heavily calcified lesions, bifurcations, left main (LM) and ostial disease, multivessel CAD, and chronic total occlusions (CTO).

In this context, CCTA is increasingly being recognized as a valuable adjunct to ICA for PCI planning. By providing comprehensive three-dimensional anatomical and morphological characterization of coronary lesions—including plaque composition, lesion length, vessel tortuosity, and calcium distribution—CCTA enables a more accurate assessment of lesion complexity before the procedure. This information can be used to optimize procedural strategy, guide upfront selection of devices and lesion preparation techniques, and anticipate technical challenges, thereby improving procedural efficiency and success rates and facilitating catheterization laboratory workflow organization in patients selected for an invasive strategy [2].

Importantly, the effective implementation of a CCTA-guided PCI workflow requires close multidisciplinary collaboration between interventional cardiologists and cardiac CT imagers, integrating non-invasive imaging findings into procedural decision-making. Nevertheless, elective PCI remains one of the few cardiovascular procedures still commonly performed without systematic pre-procedural CT imaging for planning purposes, highlighting a gap between available imaging capabilities and routine clinical practice.

This narrative review was conducted to synthesize and critically appraise the available evidence on the role of coronary computed tomography angiography (CCTA) in the planning and guidance of percutaneous coronary intervention (PCI). A comprehensive literature search was performed in PubMed, Embase, and Google Scholar, including publications available up to October 2025. Search terms combined keywords related to CCTA and PCI planning, such as “CT-guided PCI,” “FFR-CT,” “virtual PCI,” “plaque characterization,” “calcified lesions,” and “chronic total occlusions.”

Original research articles, systematic reviews, meta-analyses, expert consensus documents, and international guideline statements were considered. Particular emphasis was placed on position papers and recommendations from major scientific societies, including the Society of Cardiovascular Computed Tomography (SCCT), the Society for Cardiovascular Angiography and Interventions (SCAI), the European Society of Cardiology (ESC), and the European Association of Percutaneous Cardiovascular Interventions (EAPCI). Key clinical trials and registries evaluating CT-guided PCI strategies and CT-derived physiological assessment—such as SYNTAX III, P3, and the ongoing P4 trial—were also specifically reviewed. Table 1 provides an overview of major clinical trials and registries assessing the use of CCTA for PCI planning and guidance.

The objective of this review was to provide a concise, evidence-based overview of how CCTA contributes to pre-procedural PCI planning by defining CAD complexity, supporting individualized revascularization strategies, and complementing intraprocedural imaging and physiological assessment in contemporary interventional practice.

## 2. Atherosclerosis Analysis and Plaque Characterization: Implications for Interventional Procedures

**Figure 1:**
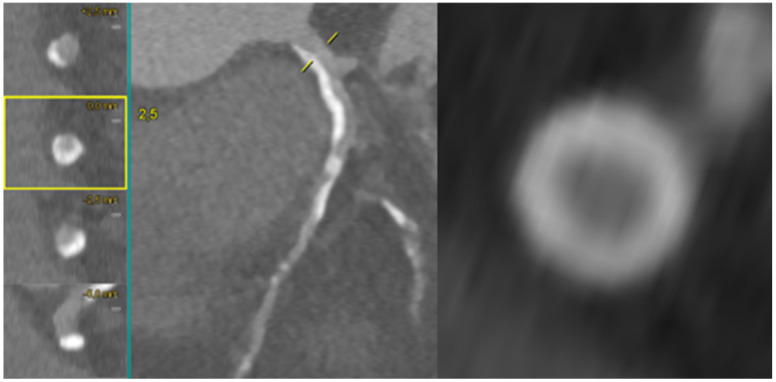
Pre-procedural CCTA assessment of a heavily calcified proximal−mid LAD lesion. Multiplanar reconstruction (MPR) and cross-sectional imaging demonstrate semi-circumferential to near-concentric calcium, consistent with a high calcific burden and supporting the need for orbital atherectomy during PCI to achieve effective lesion preparation. LAD: left anterior descending.

Non-invasive coronary evaluation with CCTA enables the comprehensive qualitative and quantitative analysis of atherosclerotic plaque features, lumen dimensions, and lesion length. These parameters translate directly into improved PCI planning and procedural outcomes. Plaques with a high calcium burden may lead to suboptimal stent expansion and apposition with an increased rate of target vessel failure, while incomplete plaque coverage or inaccurate lesion length estimation—often related to limited fluoroscopic projection—can result in inappropriate stent sizing and higher risk of target-vessel revascularization [2,3]. As shown in Figure 1, pre-procedural CCTA revealed a heavily calcified proximal–mid LAD lesion, with semi-circumferential to near-concentric calcium distribution on MPR cross-sections, indicative of a high calcific burden and ultimately requiring orbital atherectomy for adequate plaque debulking.

Beyond anatomical delineation, CCTA allows the detection of coronary plaques with high-risk features—including positive remodelling, low-attenuation core, napkin-ring sign, and spotty calcifications—which are known independent predictors of acute coronary syndrome (ACS). The capacity of CCTA to non-invasively quantify atherosclerotic burden has been validated by its strong correlation with optical coherence tomography (OCT) for plaque quantification [4]. Landmark CCTA studies have shown that low-density non-calcified plaque (LDNCP, <30 HU) occurs more frequently in patients with ACS than in those with stable CAD, and it predicts future coronary events.

Therefore, routine reporting of high-risk plaque (HRP) features and calcified plaque characterization should be incorporated into every CCTA assessment. Advanced atherosclerosis evaluation with CCTA provides detailed information on the presence, extent, and severity of calcified lesions, which can inform the use of specialized plaque modification techniques (rotational or orbital atherectomy, intravascular lithotripsy) prior to stent deployment.

**Figure 2:**
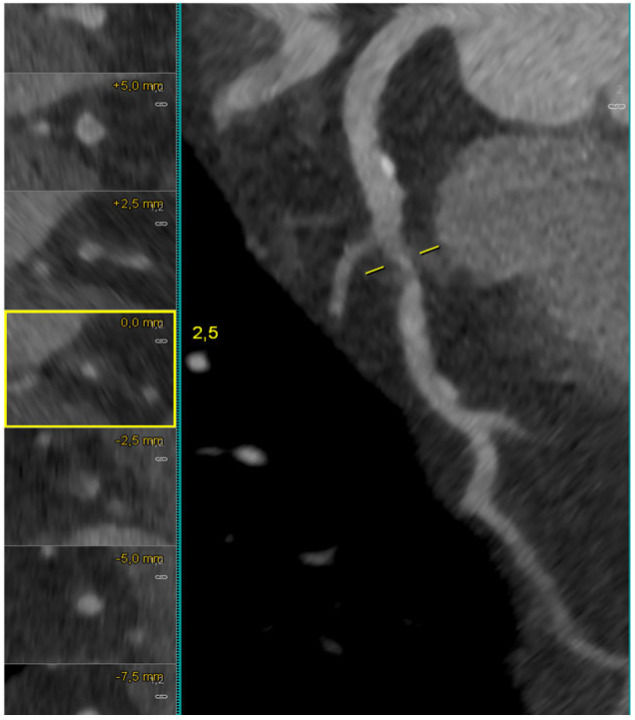
CCTA identification of a high-risk plaque in the mid LAD. Curved multiplanar reconstruction and cross-sectional images demonstrate a focal fibrolipidic plaque with homogeneous low-attenuation plaque (LAP), consistent with established high-risk features.

Moreover, CCTA features associated with plaque vulnerability have been shown to correlate with optical coherence tomography (OCT) markers of instability, such as thin-cap fibroatheroma, lipid-rich plaque, macrophage infiltration, and cholesterol crystals [4]. These features are not only linked to future cardiovascular events but are also associated with periprocedural complications such as the transient no-reflow phenomenon, which is defined as absent or slow coronary flow despite no significant residual stenosis. Specifically, lipid-rich plaques showing low attenuation and the napkin-ring sign have been strongly associated with no-reflow risk during PCI. As shown in Figure 2, CCTA identified a high-risk plaque (HRP) in the mid LAD, characterized by a homogeneous low-attenuation plaque (LAP) on curved MPR and cross-sectional views, consistent with features of plaque vulnerability.

Therefore, CCTA provides a unique opportunity to characterize atherosclerosis both morphologically and functionally. By integrating plaque quantification and vulnerability assessment into pre-PCI planning, interventional cardiologists can anticipate procedural complexity, select appropriate lesion-preparation techniques, and potentially improve long-term outcomes. Table 2 provides an overview of the main anatomical and morphological parameters derived from CCTA that are relevant for PCI planning, whereas Table 3 offers a comparative assessment of CCTA, IVUS, and OCT for plaque and calcium characterization. 

## 3. Integration of CCTA for PCI Guidance into the Cath Lab

The clinical integration of coronary CT angiography (CCTA) into the catheterization laboratory represents one of the most promising evolutions in interventional cardiology. In 2022, the Society of Cardiovascular Computed Tomography (SCCT) published its expert consensus on the pre-procedural planning of coronary revascularization, highlighting the potential of CCTA to guide PCI strategy selection and device preparation. Building on this, the 2023 European Association of Percutaneous Cardiovascular Interventions (EAPCI) emphasized CCTA’s role in non-invasive calcium burden assessment and procedural planning, while the 2024 European Society of Cardiology (ESC) guidelines for chronic coronary syndromes formally recognized the concept of “virtual PCI”, combining anatomical data from CCTA with functional insights from FFR-CT to enable comprehensive revascularization planning.

The European Bifurcation Club (EBC) further endorsed this approach, noting that CCTA guidance allows for the pre-procedural identification of optimal angiographic views, facilitates virtual physiological assessment, and supports strategic decisions such as vessel preparation, bifurcation strategy, and virtual PCI simulation. Complementing these guidelines, several ongoing clinical studies validate the clinical impact of CCTA-guided PCI: the CT COMPASS (NCT06280638) and CTS-C-CTOPCI (NCT04549896) trials compare CT-guided and angiography-guided PCI in complex disease, while CTCOMPARE (NCT06170541) and CCT-PCD-1 (NCT05551351) assess the role of next-generation photon-counting CT for procedural optimization.

The integration of anatomical and functional data from CCTA into PCI planning has been recently summarized in the Society for Cardiovascular Angiography and Interventions (SCAI)/Society of Cardiovascular Computed Tomography (SCCT) Expert Consensus Roundtable on CT-guided PCI [5]. This document outlines the essential imaging components and practical workflow for CCTA-based procedural guidance.

However, information relevant to PCI guidance—such as lesion length, plaque characteristics, calcifications, tortuosity and side-branch anatomy—can be accurately obtained from standard CCTA datasets. The use of the following four key reconstruction modalities is essential in pre-procedural planning for CCTA-guided PCI: full-volume maximum intensity projections (MIP), standard axial views, multiplanar reformations (MPR, both curved and straight), and short-axis cross-sectional images. Together, these reconstructions provide a comprehensive three-dimensional understanding of coronary anatomy, enabling optimal procedural strategy and device selection. 

The potential clinical value of CCTA in planning and guiding PCI encompasses several aspects:

1. Precise characterization of plaque morphology, burden, and composition;
2. Identification of calcified lesions requiring advanced calcium modification techniques (e.g., rotational or orbital atherectomy, intravascular lithotripsy) to optimize stent expansion and apposition;
3. Prediction of procedural success in chronic total occlusion (CTO) PCI using CCTA-derived scores;
4. Definition of procedural risk and anatomical complexity in multivessel disease or high-risk coronary anatomy (e.g., ostial and bifurcation lesions, unprotected left main disease);
5. Personalized device and material selection tailored to the revascularization strategy derived from pre-PCI CT (i.e., stents and/or balloons sizing).

Full-volume maximum intensity projection (MIP) offers a rapid overview of coronary anatomy and case complexity, including vessel dominance, coronary anomalies, tortuosity, lesion location, and calcium burden. It also supports the selection of optimal fluoroscopic angles, which is particularly relevant for ostial lesions, where precise ostial demarcation is critical to avoid geographic miss during stent deployment.

Axial reconstructions are fundamental for defining coronary ostium position and for guide catheter selection. Double-oblique imaging of the aortic root allows for the visualization of coronary take-offs and the identification of coronary anomalies. For instance, an anterior take-off of the right coronary artery on CCTA predicts insufficient support from standard Judkins catheters, prompting the use of alternative designs such as Amplatz Left. In patients with prior CABG, axial and MIP views facilitate the assessment of graft patency, ostial location, and anastomotic lesions, thus optimizing procedural planning and guiding catheter selection [5]. 3D CCTA model and C-arm co-registration represent a major advancement in intraprocedural integration. Fusion imaging overlays color-coded luminal and plaque reconstructions (based on Hounsfield units) with live angiography, enabling a synchronized 3D roadmap throughout the procedure [6]. The QAngioCT Cath Lab system (Medis Medical Imaging Systems) allows the 3D coronary tree to rotate in real time with C-arm movement, eliminating vessel foreshortening, improving wire navigation, and reducing contrast use. This hybrid view of the coronary anatomy provides dynamic visualization of lesion extent, plaque burden, and optimal landing zones.

Curved and straight multiplanar reformations (MPR) enable the precise measurement of lesion length and geometry. Short-axis cross-sections oriented perpendicular to the lumen centerline depict vessel size and plaque morphology, assisting in stent sizing selection. CCTA-based stent sizing, typically performed by modest upsizing from distal lumen diameter, has shown close agreement with intravascular imaging (OCT and IVUS) measurements [7,8].

From a procedural standpoint, CCTA-guided PCI begins with the identification of significant CAD and the characterization of lesion morphology and extent. The MLD approach (Morphology–Length–Diameter), originally developed for OCT and IVUS, can be seamlessly adapted to CCTA-based planning as follows: morphology is assessed using MIP and MPR reconstructions, lesion length from curved MPR, and lumen diameters from short-axis views acquired under vasodilator administration. Importantly, CCTA-derived stent dimensions have shown substantial agreement with OCT measurements, confirming the reliability of CT-based pre-procedural planning [8]. Table 4 compares CCTA with intravascular imaging modalities, including IVUS and OCT, while Table 5 outlines the main strengths, limitations, and practical considerations associated with CCTA-guided PCI.

The integration of CCTA into PCI planning bridges the gap between non-invasive imaging and intravascular guidance. It provides a reproducible, anatomy-based roadmap that enhances procedural precision, optimizes stent deployment, and supports risk stratification, ultimately paving the way for a hybrid model of CT-based virtual and real PCI in the modern catheterization laboratory.

### 3.1. CCTA-Based Coronary Physiology Assessment for Virtual PCI Planning: FFR-CT and Myocardial Mass at Risk

Myocardial mass quantification further enhances pre-procedural assessment. Using automated left ventricular segmentation and a Voronoi algorithm, vessel-specific myocardial mass can be calculated from the product of tissue density (1.05 g/cm^3^) and subtended volume (cm^3^). Awareness of the territory at risk assists in bifurcation PCI strategy, helping operators identify side branches that supply large myocardial territories warranting protection or rescue maneuvers during intervention. However, current software does not yet distinguish viable from non-viable myocardium.

Finally, functional assessment through FFR-CT and Virtual PCI planning bridges anatomical and physiological insights. FFR-CT applies computational fluid dynamics to quantify lesion-specific hemodynamic significance and characterize disease pattern (focal vs. diffuse) through a virtual pullback curve. The FFR-CT planner enables the simulation of various stent lengths or landing zones to predict post-PCI flow improvement and residual ischemia, which is particularly valuable in diffuse or tandem lesions.

**Figure 3:**
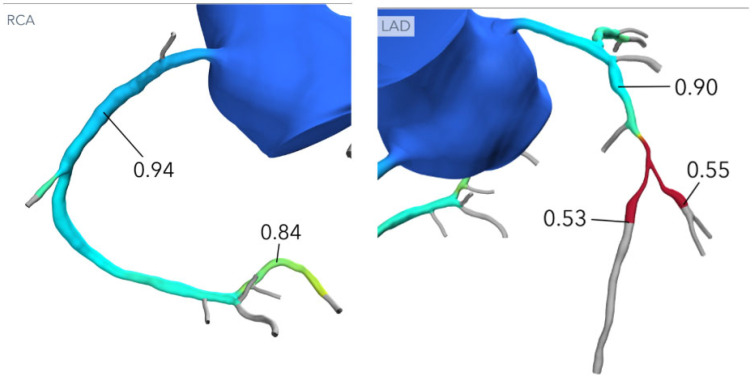
HeartFlow-derived FFR-CT assessment of an LAD–diagonal bifurcation lesion. The FFR-CT map shows markedly reduced values at the level of the LAD (0.53) and the first diagonal branch (0.55), consistent with a hemodynamically significant bifurcation lesion involving both the main vessel (MV) and the side branch (SB). In contrast, the right coronary artery demonstrates a non-significant FFR-CT value of 0.86.

Fractional Flow Reserve derived from CT (FFR-CT) extends the value of CCTA from anatomical to functional assessment, providing a comprehensive physiological map of coronary circulation. Using computational fluid dynamics (CFD) applied to coronary geometries extracted from CCTA, FFR-CT estimates mean coronary pressure distal to a stenosis divided by mean aortic pressure under simulated maximal hyperemia. This enables the non-invasive quantification of lesion-specific ischemia with accuracy comparable to invasive FFR measurements. As shown in Figure 3, FFR-CT analysis (HeartFlow model) demonstrated a hemodynamically significant bifurcation lesion in the mid LAD–first diagonal branch, with FFR-CT values <0.80, while the right coronary artery showed a non-significant value of 0.86.

FFR-CT not only identifies hemodynamically significant lesions (FFR-CT < 0.80) but also defines the functional pattern of coronary disease, distinguishing focal from diffuse atherosclerosis through a virtual pullback curve [9,10]. This characterization supports revascularization decisions (PCI vs. CABG vs. optimal medical therapy) and informs lesion selection for intervention. In the high-risk NSTE-ACS population, Meier et al. demonstrated that FFR-CT provided superior lesion-level diagnostic accuracy compared to CCTA alone (AUC 0.84 vs. 0.65; *p* < 0.01) and improved the ability to rule out hemodynamically significant stenoses, suggesting that FFR-CT may reduce unnecessary invasive angiography in this setting [11,12]. The availability of both anatomical (lumen and plaque) and physiological data in a single dataset enhances the precision of pre-procedural planning and may reduce unnecessary invasive angiography, as previously demonstrated by studies showing a higher PCI/ICA ratio when guided by FFR-CT compared to CCTA alone.

A major milestone in the integration of CT-based planning into clinical decision-making was the SYNTAX III Revolution Trial, which combined CCTA-derived coronary anatomy with FFR-CT for the non-invasive calculation of the SYNTAX Score III [13]. This score merges anatomical complexity, functional significance, and patient comorbidity to guide multidisciplinary revascularization strategies in multivessel disease, providing a non-invasive alternative roadmap for heart-team discussions.

The P3 (Precise Percutaneous Coronary Intervention Plan) study by Collet et al. further validated the concordance between CCTA-derived and OCT-derived vessel dimensions used for stent sizing, showing excellent agreement in distal landing zone measurements [14,15]. Importantly, the study demonstrated that CCTA-guided stent selection based on distal lumen diameter yielded results comparable to OCT guidance, irrespective of plaque composition.

Building upon these findings, the FFR-CT Planner (HeartFlow) enables virtual PCI simulation by modeling the expected physiological improvement following stent implantation. This tool recalculates post-PCI FFR-CT values in real time after the virtual removal of the lesion, predicting the hemodynamic benefit of revascularization. Post-PCI FFR is a validated marker of functional revascularization and an independent predictor of adverse outcomes after PCI.

In a validation study by Sonck et al., FFR-CT–predicted post-PCI values closely matched invasive FFR and post-PCI OCT metrics of luminal area, confirming the accuracy and reproducibility of the planner in both calcified and non-calcified lesions [16,17].

The clinical application of these technologies lies in optimizing stent strategy before entering the cath lab. By simulating different stent lengths and positions, operators can anticipate whether revascularization will restore physiological flow or leave diffuse pressure gradients unresolved. This is particularly relevant in tandem or diffuse lesions, where the planner can quantify the incremental benefit of treating each segment, reducing unnecessary stent length and procedural complexity. In focal CAD, PCI predicted by the planner to normalize FFR is associated with greater post-PCI FFR values and improved symptom relief, whereas diffuse disease often yields limited hemodynamic gain despite complete anatomical stent coverage.

Retrospective data also demonstrate the prognostic value of pre-PCI FFR-CT; values ≤ 0.74, in conjunction with total stent number and length, independently predict target vessel failure and revascularization [12]. The PPG (Pullback Pressure Gradient) index, originally developed from invasive physiology, can be derived virtually from FFR-CT to quantify the distribution of resistance and distinguish focal from diffuse CAD [13]. Thus, pre-PCI FFR-CT provides both diagnostic and predictive insights that guide patient selection and revascularization strategy.

The integration of FFR-CT and virtual PCI planning is a central theme of the SCAI/SCCT Roundtable 2025 Expert Consensus, which positions CCTA-based physiology as a bridge between non-invasive diagnosis and intraprocedural precision. According to the consensus, FFR-CT and the virtual planner will likely become standard components of CT-guided PCI workflows, assisting operators in determining lesion significance, optimal stent strategy, and expected post-PCI functional results within a single pre-procedural framework. FFR-CT is particularly useful in patients in whom adenosine should be avoided (e.g., severe aortic stenosis). Besides HeartFlow, other vendors are developing CCT-based physiological tools (e.g., Siemens cFFR, Canon FFR-CT, Medis QFR-CT).

A key practical limitation of FFR-CT implementation is the requirement to transfer high-quality CCTA datasets to dedicated external core laboratories for computational fluid dynamics analysis, which precludes real-time, on-table physiological assessment at the time of image acquisition. This workflow dependency introduces unavoidable processing delays, typically ranging from several hours to a full day, thereby limiting the applicability of FFR-CT in acute settings or in catheterization laboratories requiring immediate physiological decision-making. Additional barriers include cost and limited availability, as FFR-CT analysis relies on proprietary software platforms and centralized processing infrastructures that are not universally accessible across healthcare systems. These economic and logistical constraints may restrict widespread adoption, particularly in centers with limited resources or low procedural volumes. Furthermore, the requirement for optimal image quality—including adequate heart rate control, minimal motion artifacts, and precise coronary segmentation—can result in non-analyzable datasets in a subset of patients, further reducing feasibility in real-world practice.

Collectively, these factors highlight that, despite its strong diagnostic accuracy and growing clinical validation, FFR-CT currently functions best as a complementary pre-procedural planning tool rather than a replacement for intraprocedural invasive physiology. Ongoing developments in on-site computational platforms, artificial intelligence-driven segmentation, and faster processing pipelines may mitigate these limitations in the future, potentially enabling broader and more timely integration of CT-derived physiology into routine clinical workflows.

### 3.2. Bifurcation Lesions

Coronary bifurcation lesions represent one of the most challenging scenarios for percutaneous coronary intervention (PCI). Anatomical assessment is traditionally based on the Medina classification, which describes the presence of significant stenosis in three bifurcation segments and remains the reference standard recommended by the European Bifurcation Club (EBC) [18].

CCTA has emerged as a reliable and reproducible imaging modality for the pre-procedural evaluation of bifurcation anatomy. In the study by Grodecki et al., CCTA demonstrated greater reproducibility than invasive coronary angiography (ICA) for assigning Medina classes and more accurate prediction of side-branch (SB) occlusion after PCI. The discrepancy between CCTA and ICA likely reflects the superior 3D visualization and cross-sectional assessment offered by CCTA [19].

By providing a detailed depiction of plaque distribution, CCTA enables the quantification of plaque burden within the main vessel (MV), side branch (SB), and carina. These parameters improve the prediction of SB compromise, particularly in lesions with severe calcification at the bifurcation, where plaque shift from the proximal MV into the SB may cause periprocedural occlusion or deformation of bifurcation stents. The recent SCAI 2025 expert consensus emphasized the importance of CCTA-guided bifurcation analysis to optimize fluoroscopic view selection, assess take-off angle, and define the most appropriate stenting technique (e.g., provisional vs. upfront two-stent approach).

Three-dimensional reconstruction is particularly valuable in identifying plaque asymmetry, carina shift risk, and optimal projection angles. The accurate CT-based selection of fluoroscopic views minimizes foreshortening and overlap, reducing the likelihood of geographical miss or incomplete SB coverage. In this context, CT guidance assists in choosing between techniques with minimal metal overlap (e.g., T and protrusion stenting) or those offering complete SB protection (e.g., double-kissing crush) [20].

Several quantitative CT-derived risk scores have been developed to predict SB occlusion and inform bifurcation strategy.

- The V-RESOLVE score integrates five CT parameters—plaque distribution, bifurcation core stenosis, bifurcation angle, MV/SB diameter ratio, and pre-stenting SB stenosis—accurately identifying high-risk cases (≥17 points) with a markedly increased incidence of SB occlusion (18.6% vs. 3.8%) [21].
- The CT bifurcation score proposed by Lee et al. incorporates plaque type and location (calcified or necrotic) and the MV-to-SB vessel area ratio (>4.3). A score ≥1 predicts a significantly higher likelihood of SB occlusion and may justify upfront protection or a two-stent strategy, whereas a score of 0 indicates low risk and supports a provisional approach [22].

Subsequent analyses from the CT-PRECISION registry confirmed that non-calcified plaque burden within 5 mm of the SB ostium is the strongest predictor of SB occlusion during bifurcation PCI. Incorporating quantitative plaque data into visual assessment further enhances predictive accuracy [23].

**Figure 4:**
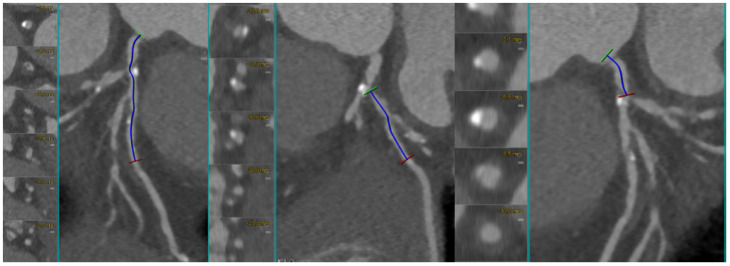
CCTA assessment of a complex true bifurcation lesion (Medina 1.1.1). Curved MPR and cross-sectional views allow for the accurate measurement of lesion length in both the main branch (MB) and side branch (SB), enabling precise landing zone definition and stent length selection. CCTA also provides an exact estimation of the distance between the left main (LM) ostium and the carina, which is essential for selecting the appropriate balloon length for the proximal optimization technique (POT). Abbreviations: LM, left main; LAD, left anterior descending; LCx, left circumflex; MB, main branch; SB, side branch; POT, proximal optimization technique.

In summary, CCTA enables the comprehensive, quantitative assessment of bifurcation anatomy and plaque distribution, offering insights unattainable with conventional angiography. By combining anatomical, morphologic, and quantitative data, CCTA-derived risk models guide personalized bifurcation strategies, improve procedural planning, and may ultimately reduce periprocedural complications. Therefore, CCTA enables more accurate bifurcation analysis—including precise MV-SB angle assessment—and thus helps identify the most suitable two-stent strategy, including DK-crush, Culotte or TAP technique. As shown in Figure 4, CCTA provided a detailed anatomical assessment of a complex true LM–LAD–LCx bifurcation lesion (Medina 1.1.1). The dataset allowed for the accurate measurement of lesion length in both the main and side branches, supporting the precise identification of landing zones and appropriate stent sizing. Furthermore, CCTA enabled the exact definition of the distance between the LM ostium and the carina, guiding the optimal balloon length selection for POT.

**Figure 5:**
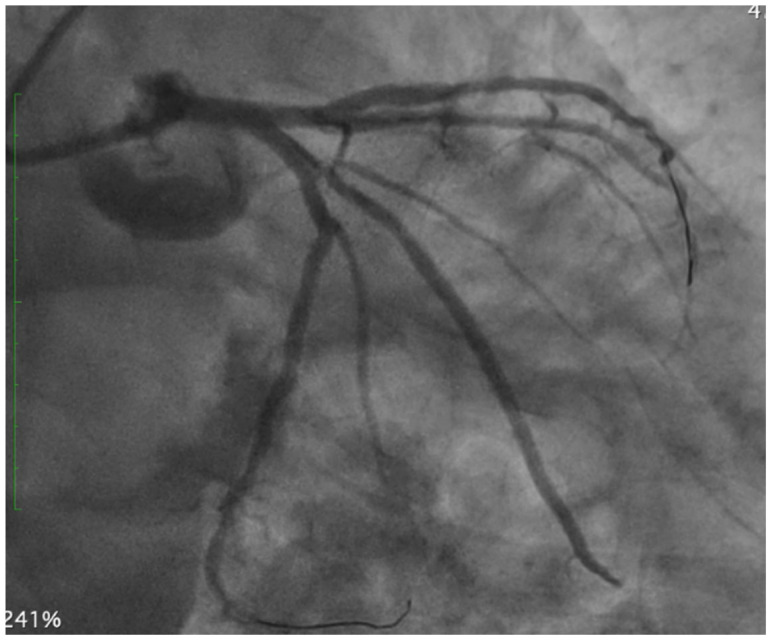
Final angiographic result. Angiography shows optimal lumen restoration following T-and-protrusion (TAP) stenting of the left main (LM) bifurcation involving the left anterior descending (LAD) and left circumflex (LCx). Abbreviations: PCI, percutaneous coronary intervention; LM, left main; LAD, left anterior descending; LCx, left circumflex; TAP, T-and-protrusion.

As shown in Figure 5, the final angiographic result demonstrates the successful treatment of the bifurcation lesion using a two-stent strategy (TAP technique), with optimal stent expansion, complete restoration of luminal patency, and final TIMI 3 flow in both the LAD and LCx.

### 3.3. Aorto-Ostial Lesions

Aorto-ostial lesions pose particular technical challenges during PCI due to difficulty in defining the true ostium and the risk of longitudinal geographic miss. CCTA is particularly advantageous in this setting, allowing for the precise visualization of the lesion’s relationship to the aortic wall and accurate measurement of the vessel take-off angle. The pre-procedural CT-guided selection of optimal fluoroscopic projections reduces foreshortening and overlap, improving stent alignment and deployment accuracy.

The double-S curve method, described by Kocka et al., provides the CT-based determination of optimal viewing angles for left and right coronary ostia, minimizing anatomic distortion and ensuring complete lesion coverage. Moreover, ostial RCA lesions frequently have extreme take-off angles that are difficult to appreciate on ICA and clearly delineated on CCTA, often corresponding to an LAO-cranial projection. Accurate stent length selection is crucial, as inadequate longitudinal coverage has been associated with up to a threefold increase in target-vessel revascularization.

### 3.4. Heavily Calcified Lesions

Percutaneous coronary intervention (PCI) of calcified coronary stenosis (CCS) is associated with lower procedural success and a higher risk of both early and late complications [24]. Calcified plaques reduce vessel compliance, impair device deliverability, and hinder symmetric balloon expansion, leading to stent underexpansion, malapposition, and suboptimal acute lumen gain compared to non-calcified lesions. These limitations are also associated with a higher incidence of dissection, restenosis, and target-vessel failure. Adequate lesion preparation and calcium modification are therefore essential to achieve optimal stent expansion and improve outcomes. Ablation or fracture of calcified plaques (via rotational/orbital atherectomy, laser, or intravascular lithotripsy) increases vessel compliance, enabling proper stent deployment.

A landmark study by Sekimoto et al. demonstrated that a per-lesion coronary calcium score derived from multidetector CCTA independently predicts the need for rotational atherectomy during the PCI of calcified lesions. In patients with stable angina, a regional calcium score > 453 predicted rotablation with 93% sensitivity and 88% specificity, outperforming QCA-derived stenosis and the circumferential calcium arc, while a calcium arc ≥ 270° was also highly predictive of the need for advanced calcium modification strategies (71% sensitivity, 97% specificity) [25].

Intravascular imaging plays a central role in the management of heavily calcified coronary lesions by guiding the need for plaque modification prior to stent implantation. OCT- and IVUS-based calcium scoring systems have demonstrated that calcium arc, thickness, and length are strong predictors of stent underexpansion and can identify lesions requiring debulking or advanced calcium modification strategies before PCI [26,27]. In this context, automated 3D MIP reconstructions from CCTA allow for the rapid appraisal of lesion complexity by highlighting potentially undilatable or uncrossable segments, while straight MPR and cross-sectional reconstructions enable the precise evaluation of calcium length, localization, arc, thickness, and density, which are parameters that closely correlate with intravascular imaging–derived calcium scores.

From a planning perspective, prospective studies on CCTA-guided calcium modification are still limited, yet evidence from intravascular imaging can be extrapolated to support the clinical relevance of CCTA-derived calcium metrics. Three-dimensional CT imaging enhances the understanding of calcified plaque morphology, distribution, and proximity to critical anatomical landmarks (e.g., bifurcations, ostial segments). This spatial insight enables the anticipation of procedural challenges—such as device delivery issues or guide support limitations—and early planning for the use of guide extensions, microcatheters, or calcium modification devices.

Calcium density (measured in Hounsfield units) provides a surrogate for mechanical hardness and response to therapy. High-density calcium (>800 HU) is generally resistant to balloon angioplasty and may necessitate intravascular lithotripsy or atherectomy, whereas low-density calcium may be adequately treated with non-compliant balloon dilatation. CCTA automatically quantifies calcium density for each cross-sectional image, offering a reproducible and objective measure of lesion hardness.

Qualitative plaque analysis through CCTA allows for the evaluation of calcification extension (arc and length), density, and eccentricity, which is critical for selecting the optimal debulking technique. Circumferential and long calcifications are associated with a higher risk of under-expansion and poor stent apposition. Despite potential blooming artifacts leading to calcium overestimation, cross-sectional CCTA remains a robust predictor of calcium severity. The analysis of calcified plaque characteristics predictive of the need for advanced calcium modification or debulking techniques has demonstrated good concordance between coronary CT angiography (CCTA) and intravascular imaging modalities, namely intravascular ultrasound (IVUS) and optical coherence tomography (OCT), which are considered the reference standards for plaque assessment. Quantitative and qualitative parameters derived from CCTA—such as calcium arc, thickness, and length—show strong correlations with corresponding measurements obtained from IVUS and OCT, supporting the reliability of CT-based plaque characterization for procedural planning and lesion preparation.

Several comparative studies have validated these findings, confirming that CCTA can accurately quantify calcium burden and morphologic features in agreement with invasive intravascular imaging. Conte et al. demonstrated that plaque quantification by CCTA exhibits excellent concordance with IVUS across different CT scanner generations [28]. Similarly, Monizzi et al. reported a strong correlation between calcium burden quantified by CCTA and OCT measurements, emphasizing the reproducibility and clinical applicability of CT-based calcium assessment [29]. Matsumoto et al. further confirmed the robustness of standardized volumetric plaque quantification by CCTA compared to IVUS [7]. More recent investigations have refined these insights as follows: Okutsu et al. established that coronary calcium thickness can be reliably estimated on CCTA using OCT as a reference [30], while Kurogi et al. demonstrated that combined CT–OCT evaluation enhances the precision of calcium characterization and may improve procedural strategy selection for calcified coronary lesions [31].

**Figure 6:**
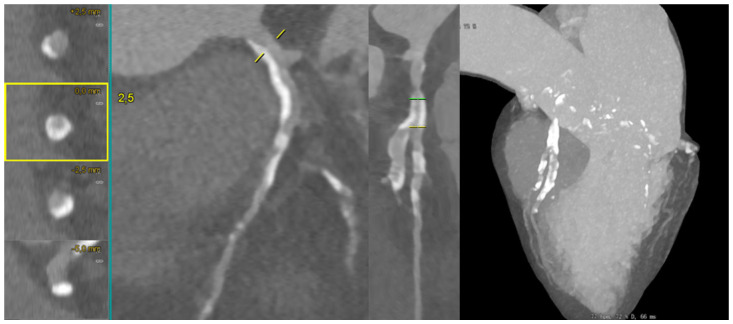
CCTA evaluation of a heavily calcified LAD–D1 bifurcation lesion. Long-axis MPR reconstruction of the left anterior descending artery (LAD) shows an extended calcified plaque involving the proximal and mid segments, with an additional MPR view illustrating severe calcification across the LAD–first diagonal (D1) bifurcation. The MIP CCTA reconstruction further highlights the complex and heavily calcified morphology of the lesion. Abbreviations: LAD, left anterior descending; D1, first diagonal; MPR, multiplanar reconstruction; MIP, maximum intensity projection; CCTA, coronary computed tomography angiography.

Figure 6 illustrates a calcified LAD–D1 bifurcation lesion, where long-axis and cross-sectional MPR views demonstrate semi-circumferential calcium distribution, allowing for the semiquantitative assessment of the calcific burden. The complementary MIP reconstruction highlights the extent of calcification across the bifurcation segment.

The mechanism of successful stent expansion in calcified lesions is the fracture of the calcium plate. While rotational or orbital atherectomy are the most effective tools for uncrossable lesions, their efficacy depends on the relationship between wire bias, calcium distribution, and lumen geometry. Predicting this interaction is increasingly possible using 3D CT reconstructions as follows:

- Wire bias prediction—the tendency of the guidewire to remain in contact with one side of the vessel wall—can be inferred from the spatial orientation of the calcified arc;
- Overlay of the predicted wire path on the calcium density map allows for the estimation of device–calcium contact zones, guiding the choice between ablation or fracture-based modification tools.

This mechanistic insight from CCTA—combining plaque morphology, calcium density, and 3D geometry—supports the individualized selection of lesion preparation techniques and anticipates procedural risks. Collectively, these features underscore CCTA’s potential as a non-invasive intravascular roadmap, capable of preemptively identifying complex calcified lesions and guiding optimal calcium modification strategy.

### 3.5. Chronic Total Occlusion (CTO)

Chronic total occlusions (CTO) represent one of the most technically challenging scenarios in percutaneous coronary intervention (PCI), owing to longer procedural duration, increased contrast exposure, and lower procedural success rates compared to non-occlusive coronary lesions. Randomized clinical trials such as EuroCTO, IMPACTOR-CTO, and COMET-CTO have demonstrated that CTO PCI, when performed in appropriately selected patients, is associated with sustained clinical benefit and acceptable long-term safety compared to optimal medical therapy, reinforcing the clinical relevance of CTO revascularization [32,33,34].

Given the complexity of CTO interventions, accurate pre-procedural anatomical characterization is essential to optimize procedural strategy and improve outcomes. In this context, coronary computed tomography angiography (CCTA) has emerged as a valuable non-invasive imaging modality, offering comprehensive three-dimensional visualization of CTO morphology. Compared to invasive coronary angiography, CCTA provides superior assessment of occlusion length, vessel course, calcification burden, and distal vessel anatomy, all of which are critical determinants of guidewire crossing success and procedural complexity [35,36].

### 3.6. CTO Scoring Systems Derived from CCTA

Several scoring systems have been developed to predict CTO PCI difficulty and procedural success. The J-CTO score, originally derived from angiographic parameters, remains the traditional reference and incorporates proximal cap ambiguity, calcification, vessel bending > 45°, occlusion length > 20 mm, and prior failed attempt. Importantly, Fujino et al. demonstrated that J-CTO scoring derived from CCTA provides predictive accuracy comparable to angiography-based assessment, supporting the use of CT as an alternative modality for CTO risk stratification [37].

Beyond angiography-derived scores, dedicated CCTA-based models have been developed to exploit the superior spatial resolution of CT imaging. The CT-RECTOR score, derived from a multicenter registry, integrates CT-specific morphological variables such as blunt stump morphology, severe calcification, multiple occlusions, and occlusion length, improving the prediction of guidewire crossing time and procedural difficulty [38]. Additional studies confirmed that CCTA-derived anatomical parameters independently predict procedural success and wire crossing efficiency [39].

More comprehensive scoring systems have further refined prognostic accuracy. The incorporation of quantitative high-density plaque metrics into CT-based models has been shown to enhance the prediction of guidewire crossing time [40]. The CASTLE score, obtained from CCTA, accurately predicts early wire crossing within 30 min [41], while the RECHARGE and RECHARGE-CTO scores integrate detailed anatomical and plaque-related information to predict technical success in hybrid CTO PCI strategies [42,43]. Collectively, these scores underscore the incremental value of CCTA over conventional angiography in pre-procedural CTO assessment. Table 6 summarizes CCTA-based scoring systems used to predict procedural complexity and technical success in CTO PCI.

### 3.7. CT-Derived Predictors of Procedural Difficulty

CCTA enables the detailed evaluation of morphological features associated with CTO PCI complexity. Proximal cap ambiguity—defined as uncertainty regarding the occlusion entry point—remains a major determinant of procedural success. CCTA allows for the precise delineation of stump morphology and its relationship with adjacent side branches, facilitating the accurate identification of the entry site and selection of optimal fluoroscopic projections. Occlusion length and calcification burden are additional key predictors of difficulty. Longer occlusions (>20 mm) and extensive or circumferential calcification significantly reduce the likelihood of successful guidewire crossing and are optimally visualized using curved and straight multiplanar reformations and short-axis CT views. Vessel tortuosity and distal vessel quality can also be assessed three-dimensionally, supporting appropriate guidewire and microcatheter selection.

Systematic reviews and meta-analyses have confirmed the incremental prognostic value of pre-procedural CCTA in CTO PCI. In particular, the combined assessment of occlusion length, calcium burden, and vessel tortuosity provides the highest predictive accuracy for procedural success and failure. Recent studies further demonstrated that CCTA improves the prediction of failed PCI attempts and should be considered an integral component of CTO planning [44,45,46].

### 3.8. Integration into Strategy Planning and Procedural Guidance in CTO

Beyond risk stratification, CCTA plays a central role in procedural strategy planning and intraprocedural guidance for CTO PCI. CCTA-derived anatomical models enable the pre-procedural simulation of guidewire trajectories and identification of potential re-entry zones. Three-dimensional curved and straight multiplanar reconstructions assist in defining optimal fluoroscopic viewing angles, while cross-sectional calcium assessment predicts balloon uncrossability and the potential need for atherectomy.

The integration of CCTA datasets into hybrid imaging platforms allows for real-time CT–fluoroscopy co-registration during PCI. This approach provides the intraprocedural visualization of the occluded segment aligned with live angiography, improving wire targeting accuracy, facilitating dissection–reentry techniques, and reducing contrast use, particularly in complex CTO anatomy [47,48]. When distal vessel visualization is limited on angiography, CCTA reconstruction offers a detailed roadmap of the distal lumen and side branches, enabling anticipation of potential retrograde collateral pathways. From a clinical perspective, by providing comprehensive morphological and spatial information, CCTA enhances procedural predictability, guides wire and device selection, and facilitates individualized CTO PCI strategy planning. Evidence from randomized trials and large registries, including EuroCTO, IMPACTOR-CTO, and COMET-CTO, collectively confirms the safety and efficacy of CTO PCI and highlights the potential role of CCTA as a standard adjunct for optimizing CTO intervention planning.

## 4. Future Perspectives

The ongoing P4 (Precise PCI Planning) trial (NCT05253677) represents a key step toward clinical validation of CCTA-guided PCI. P4 is an investigator-initiated, multicenter, randomized study with a non-inferiority design, comparing a coronary CT-guided PCI strategy with an intravascular ultrasound (IVUS)-guided PCI strategy in patients with an indication for percutaneous revascularization. After identification of a significant coronary stenosis, patients are randomized to either CT-guided or IVUS-guided PCI, with both strategies performed according to a standardized protocol. The CT-guided approach integrates comprehensive morphological and functional assessment, including plaque characteristics, vessel geometry, and CT-derived physiological information, while IVUS guidance represents the current intravascular imaging reference standard. Post-PCI fractional flow reserve is systematically measured to provide an objective functional assessment of revascularization. Patients are followed during hospitalization, at 30 days, 12 months, and annually up to 5 years. Preliminary data from the P3 registry demonstrated a high concordance between CCTA- and OCT-derived stent sizing, associated with favorable acute procedural outcomes, providing the rationale for P4. The trial is expected to determine whether CT-guided PCI is non-inferior to IVUS-guided PCI in terms of procedural success and clinical outcomes, thereby supporting the role of CCTA as a validated platform for PCI planning and guidance.

The diagnostic performance of photon-counting detector CT (PCCT) for the detection of coronary in-stent restenosis has been confirmed in recent studies, demonstrating accuracy comparable to invasive coronary angiography and superior lumen visualization, particularly in small-diameter and complex stents [49,50,51]. These findings build upon earlier experience with multidetector CT, which has already shown acceptable diagnostic reliability for the assessment of stent patency, albeit with limitations related to blooming artifacts and spatial resolution [52]. Advances in artificial intelligence (AI) and machine learning now allow for automated plaque segmentation, calcium quantification, and lumen reconstruction, providing real-time procedural insights and reducing operator variability. Moreover, hybrid fusion systems integrating CT datasets with intraprocedural fluoroscopy are transforming CCTA from a static diagnostic tool into an active guidance modality, enabling precise three-dimensional co-registration between coronary anatomy and live angiography.

Beyond anatomical detail, CT-based functional imaging—including FFR-CT, iFR-CT, and virtual pullback indices—extends the utility of CCTA into the physiological domain, refining lesion selection and optimizing strategy in diffuse or borderline disease. As these technologies converge, CT-guided PCI is increasingly positioned as a routine component of interventional workflow. In the near future, procedural planning may be conducted entirely in silico, allowing operators to simulate stent deployment, predict hemodynamic improvements, and anticipate potential complications before entering the catheterization laboratory.

AI-enabled CCTA platforms allow for automated plaque characterization and physiological modeling, with recent multicenter data showing strong agreement with intravascular imaging and high reproducibility, thereby supporting their role in CT guided PCI planning [53]. CCTA has the potential to close the gap between diagnosis and intervention, promoting safer, faster, and more effective revascularization. The results of the P4 trial will be pivotal in validating this transition and may establish CT-guided PCI as a new standard of care, one in which imaging does not merely follow the procedure but actively drives it.

## 5. Conclusions

The progressive integration of coronary computed tomography angiography (CCTA) into interventional cardiology workflows marks a paradigm shift toward non-invasive, image-based precision revascularization. Technological innovation and growing clinical evidence are rapidly redefining the role of CT in the planning, execution, and follow-up of percutaneous coronary intervention (PCI). Coronary CT angiography has thus evolved from a purely diagnostic tool into a comprehensive platform that unifies anatomical, morphological, and physiological data to guide percutaneous coronary intervention. Evidence from validation studies, expert consensus, and emerging clinical trials consistently demonstrates that CT-guided PCI enhances procedural planning, anticipates technical challenges, and supports personalized revascularization strategies. In this emerging paradigm, CT-guided PCI will not merely complement invasive imaging, it will redefine it, inaugurating a new era of precision-guided coronary intervention in which imaging truly drives therapeutic decision-making.

## Figures and Tables

**Table 1:** Major Clinical Trials and Registries Evaluating CCTA-Guided PCI. This table summarizes landmark and contemporary clinical studies assessing the utility of CCTA, CT-derived physiology, and CT–fluoroscopy fusion for planning and guiding PCI. Included studies encompass randomized controlled trials, prospective registries, and ongoing investigations that evaluate procedural strategy, stent sizing accuracy, procedural efficiency, and clinical outcomes using CT-guided approaches. CCTA = coronary computed tomography angiography; PCI = percutaneous coronary intervention; LMS = left main stem; CAD = coronary artery disease; FFR-CT = fractional flow reserve derived from computed tomography; CT = computed tomography; CTO = chronic total occlusion; RCT = randomized controlled trial.

Study/Registry	Design and Population	CT-Based Tools	Key Findings	Clinical Implications
SYNTAX III Revolution	Prospective randomized; multivessel/LMS CAD	CCTA, FFR-CT, non-invasive SYNTAX score	High agreement between CT-based and angiography-based heart-team decisions	Supports fully CT-based revascularization planning
P3 (Precise PCI Plan)	Prospective multicenter; CCTA vs. OCT for stent sizing	CCTA lumen and landing zone measurements	Excellent concordance with OCT-derived stent dimensions	Validates CCTA for stent sizing
P4 Trial (Ongoing)	Large RCT; CT-guided vs. angiography-guided PCI	CCTA, FFR-CT, virtual PCI	Results pending; primary endpoint procedural success	May establish CT-guided PCI as standard of care
CT-COMPASS	Prospective complex CAD population	CCTA morphology, calcium metrics, FFR-CT	Ongoing evaluation of procedural efficiency	Clarifies benefits in complex lesions
CTS-C-CTOPCI	Randomized trial for CTO PCI	CCTA-based CTO morphology	Reduced procedure time; improved crossing efficiency (preliminary)	Supports CT-guided CTO planning
IMPACTOR-CTO	Prospective registry for CTO PCI	CCTA morphology, plaque burden	Improves prediction of success and crossing time	Strengthens CT role in CTO strategy
COMET-CTO	Multicenter registry; CT–fluoro fusion	CT–fluoroscopy co-registration	Improved wire navigation; reduced contrast use	Demonstrates feasibility of hybrid CT-guided PCI

**Table 2:** CCTA-Derived Anatomical and Morphological Parameters Relevant for PCI Planning. This table outlines the primary anatomical, morphological, and physiological variables extracted from CCTA that influence pre-procedural PCI planning. Parameters include lumen dimensions, plaque composition, calcium metrics, tortuosity, bifurcation anatomy, ostial morphology, and CT-derived physiological indices. These features support stent sizing, lesion preparation, strategy selection, and prediction of procedural complexity. CCTA = coronary computed tomography angiography; PCI = percutaneous coronary intervention; MPR = multiplanar reconstruction; LAP = low-attenuation plaque; SB = side branch; HU = Hounsfield units; FFR-CT = fractional flow reserve derived from computed tomography.

Domain	CCTA Parameter	Clinical Meaning	Impact on PCI Planning
Lumen/Vessel Geometry	Minimal lumen diameter, reference diameters, remodeling index	Quantifies stenosis severity, identifies positive remodeling	Stent sizing, landing zone selection
Lesion Length	Curved MPR-derived length	Accurate 3D length measurement	Minimizes geographic miss
Plaque Composition	Calcified, non-calcified, LAP, napkin-ring sign, spotty calcium	Predicts vulnerability and periprocedural risk	Anticipates no-reflow, guides need for atherectomy
Calcium Burden	Arc, length, depth, density (HU)	Assesses mechanical resistance	Predicts need for calcium modification
Tortuosity	Centerline-derived angulation or multi-segment bends	Influences device deliverability	Supports choice of guide catheter, extensions, microcatheters
Side Branch Anatomy	SB angle, diameter, plaque distribution	Predicts SB occlusion risk	Guides provisional vs. two-stent strategy
Ostial Analysis	Take-off angle, aorto-ostial geometry, ostial calcium	Identifies risk of geographic miss	Determines optimal fluoroscopic projections
CT-Derived Physiology	FFR-CT, virtual pullback, myocardial mass at risk	Defines lesion significance and disease pattern	Optimizes lesion selection, virtual PCI strategy

**Table 3:** Comparative Assessment of CCTA, IVUS, and OCT for Plaque and Calcium Characterization. This table compares the diagnostic performance and technical characteristics of CCTA, IVUS, and OCT for evaluating coronary plaque burden, calcium distribution, lesion morphology, vulnerability features, and vessel sizing. Strengths and limitations of each modality are summarized alongside concordance findings from validation studies. CCTA = coronary computed tomography angiography; IVUS = intravascular ultrasound; OCT = optical coherence tomography; LAP = low-attenuation plaque; PR = positive remodeling; NRS = napkin-ring sign; TCFA = thin-cap fibroatheroma; PCCT = photon-counting computed tomography.

Parameter	CCTA	IVUS	OCT	Concordance/Notes
Plaque burden	Quantitative, good correlation with IVUS	Gold standard	Near-histologic	High CCTA–IVUS concordance
Calcium arc	Accurate but blooming exaggerates	Moderate	Excellent	CT correlates well with OCT for arc
Calcium thickness	Estimable; improved with PCCT	Moderate	Best modality	OCT superior; CCTA moderately accurate
Calcium length	Accurate on long-axis MPR	Accurate	Accurate	Good CCTA–OCT/IVUS agreement
Plaque type	Good sensitivity for LAP/NR	Moderate	Excellent microstructure	OCT best; CT useful screening
Stent sizing	Accurate for distal lumen dimensions	Acceptable	Best for expansion	P3: CCTA ≈ OCT for sizing
Vulnerability features	Detects LAP, PR, NRS	Limited	Best for TCFA	Complementary modalities

**Table 4:** Comparative assessment of CCTA and intravascular imaging modalities (IVUS and OCT) for PCI planning. Multiple validation studies have demonstrated a good agreement between CCTA-derived plaque and calcium metrics and intravascular imaging (IVUS, OCT), which remains the reference standard for intraprocedural assessment. While IVUS and OCT offer superior spatial resolution and direct visualization of calcium burden and lesion length, CCTA provides a comprehensive, non-invasive evaluation of plaque burden and calcium distribution before the procedure, enabling effective pre-procedural planning and anticipation of lesion complexity. OCT = optical coherence tomography; IVUS = intravascular ultrasound.

Imaging Modality	Technical Characteristics	Plaque and Calcium Assessment	Role in PCI Planning	Clinical Implications
CCTA	Non-invasive imaging with whole-coronary tree coverage and moderate spatial resolution	Semi-quantitative plaque burden; reliable assessment of calcium arc, length, distribution, and density (HU-based); indirect estimation of calcium thickness	Pre-procedural lesion characterization, stent sizing, and anticipation of lesion complexity and need for calcium modification	Enables comprehensive non-invasive PCI planning, improves procedural efficiency, and supports upfront strategy selection
IVUS	Invasive imaging with moderate spatial resolution and deep vessel wall penetration	Quantitative plaque burden and vessel remodeling; limited ability to assess calcium thickness	Intraprocedural guidance for stent sizing and optimization; reference standard for plaque quantification	Guides device selection and confirms adequate stent expansion during PCI
OCT	Invasive imaging with high spatial resolution and limited tissue penetration	Precise assessment of calcium arc, thickness, and length; detailed plaque microstructure visualization	Intraprocedural decision-making, particularly for calcium modification strategies and stent optimization	Identifies lesions at high risk of stent underexpansion and guides debulking techniques

**Table 5:** Strengths, Limitations, and Practical Considerations of CCTA-Guided PCI. This table provides a structured overview of the principal advantages, technical limitations, and workflow considerations associated with the use of CCTA for PCI planning and guidance. It incorporates factors related to image acquisition, calcium visualization, plaque evaluation, physiological assessment, CTO mapping, and bifurcation/ostial strategy optimization. CCTA = coronary computed tomography angiography; PCI = percutaneous coronary intervention; HRP = high-risk plaque; FFR-CT = fractional flow reserve derived from computed tomography; CTO = chronic total occlusion.

Strengths	Limitations	Practical Considerations
Comprehensive 3D anatomical assessment	Image quality dependent (HR, BMI, motion)	Optimizes CT acquisition protocols
Accurate lesion length and diameter	Calcium blooming overestimates calcification	Photon-counting CT reduces blooming
Plaque characterization and HRP detection	Limited microstructure resolution	Supplement with OCT when needed
Pre-procedural physiology (FFR-CT)	Requires core-lab processing	On-site computation emerging
Virtual PCI and stent simulation	Not real-time yet	Useful for diffuse/tandem lesions
CTO roadmap and scoring	Limited collateral visualization	Use fusion systems when available
Optimal projection planning for ostial/bifurcation	Radiation exposure (low with modern CT)	Modern scanners minimize dose

**Table 6:** CCTA-Based Scoring Systems for Predicting CTO PCI Complexity and Success. This table summarizes validated and emerging CCTA-derived scoring systems used to predict procedural difficulty, guidewire crossing success, and overall strategy planning in chronic total occlusion (CTO) PCI. Components, intended applications, and predictive performance are presented for each score. CCTA = coronary computed tomography angiography; CTO = chronic total occlusion; PCI = percutaneous coronary intervention.

Score	Components	Purpose	Predictive Value
CT-RECTOR	Blunt stump, severe calcification, bending > 45°, occlusion length > 20 mm, multiple occlusions	Predict procedural success/time	Better discrimination than J-CTO
CASTLE	Calcification, age, stump morphology, tortuosity, length, prior attempts	Predict wire crossing ≤30 min	Strong registry validation
RECHARGE-CTO	CT morphology + angiographic complexity	Predict success of hybrid strategy	Improved prediction vs. angiography alone
IMPACTOR-CTO Model	CT plaque burden, calcification, vessel course	Predict crossing success and time	Validated multicenter model
COMET-CTO CT–Fluoro Fusion	CT anatomy with real-time fluoroscopy	Intraprocedural guidance	Improves navigation and reduces contrast

## Data Availability

This study did not generate or analyze new datasets. Therefore, data sharing is not applicable.

## Abbreviations

The following abbreviations are used in this manuscript:

CCTA	Coronary CT Angiography
PCI	Percutaneous Coronary Intervention
SB	Side Branch
MV	Main Vessel
MPR	MultiPlanar Reconstruction
MIP	Maximum Intensity Projection
HU	Hounsfield Unit
QCA	Quantitative Coronary Analysis
AI	Artificial Intelligence
LAP	Low Attenuation Plaque
HRP	High Risk Plaque
LDNP	Low Density Non-Calcified Plaque
LM	Left Main
LAD	Left Anterior Descending
LCX	Left Circumflex
RCA	Right Coronary Artery
OCT	Optimal Coherence Tomography
IVUS	Intravascular Ultrasound
ACS	Acute Coronary Syndrome
CTO	Chronic Total Occlusion
CCS	Chronic Coronary Syndrome
CAD	Coronary Artery Disease
FFR	Fractional Flow Reserve

## References

[1] ESC Scientific Document Group (2024). 2024 ESC Guidelines for the management of chronic coronary syndromes. Eur. Heart J..

[2] Andreini D., Collet C., Leipsic J., Nieman K., Bittencurt M., De Mey J., Buls N., Onuma Y., Mushtaq S., Conte E. (2022). Pre-procedural planning of coronary revascularization by cardiac computed tomography: An expert consensus document of the Society of Cardiovascular Computed Tomography. EuroIntervention.

[3] Tzimas G., Gulsin G.S., Takagi H., Mileva N., Sonck J., Muller O., Leipsic J.A., Collet C. (2022). Coronary CT angiography to guide percutaneous coronary intervention. Radiology.

[4] Kinoshita D., Suzuki K., Usui E., Hada M., Yuki H., Niida T., Minami Y., Lee H., McNulty I., Ako J. (2024). High-risk plaques on coronary computed tomography angiography: Correlation with optical coherence tomography. JACC Cardiovasc. Imaging.

[5] Sandoval Y., Leipsic J.A., Collet C., Ali Z.A., Azzalini L., Barbato E., Cavalcante J.L., Costa R.A., Garcia-Garcia H.M., Jones D.A. (2025). Coronary computed tomography angiography to guide percutaneous coronary intervention: Expert opinion from a SCAI/SCCT roundtable. J. Soc. Cardiovasc. Angiogr. Interv..

[6] Collet C., Sonck J., Leipsic J., Monizzi G., Buytaert D., Kitslaar P., Andreini D., De Bruyne B. (2021). Implementing coronary computed tomography angiography in the catheterization laboratory. JACC Cardiovasc. Imaging.

[7] Matsumoto H., Watanabe S., Kyo E., Tsuji T., Ando Y., Otaki Y., Cadet S., Gransar H., Berman D.S., Slomka P. (2019). Standardized volumetric plaque quantification and characterization from coronary CT angiography: A head-to-head comparison with invasive intravascular ultrasound. Eur. Radiol..

[8] Ko B., Ohashi H., Mizukami T., Sakai K., Sonck J., Nørgaard B.L., Maeng M., Jensen J.M., Ihdayhid A., Tajima A. (2024). Stent sizing by coronary CT angiography compared with optical coherence tomography. J. Cardiovasc. Comput. Tomogr..

[9] Wang Z., Tang C., Zuo R., Zhou A., Xu W., Zhong J., Xu Z., Zhang L. (2024). Pre-PCI CT-FFR predicts target vessel failure after stent implantation. J. Thorac. Imaging.

[10] Collet C., Sonck J., Vandeloo B., Mizukami T., Roosens B., Lochy S., Argacha J.-F., Schoors D., Colaiori I., Di Gioia G. (2019). Measurement of hyperemic pullback pressure gradients to characterize patterns of coronary atherosclerosis. J. Am. Coll. Cardiol..

[11] Meier D., Skalidis I., De Bruyne B., Qanadli S.D., Rotzinger D., Eeckhout E., Collet C., Muller O., Fournier S. (2020). Ability of FFR-CT to detect the absence of hemodynamically significant lesions in patients with high-risk NSTE-ACS admitted in the emergency department with chest pain: Study design and rationale. Int. J. Cardiol. Heart Vasc..

[12] Meier D., Andreini D., Cosyns B., Skalidis I., Storozhenko T., Mahendiran T., Assanelli E., Sonck J., Roosens B., Rotzinger D.C. (2025). Usefulness of FFR-CT to exclude haemodynamically significant lesions in high-risk NSTE-ACS. EuroIntervention.

[13] Andreini D., Modolo R., Katagiri Y., Mushtaq S., Sonck J., Collet C., De Martini S., Roberto M., Tanaka K., Miyazaki Y. (2019). SYNTAX III REVOLUTION Investigators. Impact of fractional flow reserve derived from coronary computed tomography angiography on heart team treatment decision-making in patients with multivessel coronary artery disease. Circ. Cardiovasc. Interv..

[14] Andreini D., Mushtaq S., Conte E., Mei M., Nicoli F., Melotti E., Pompilio G., Pepi M., Bartorelli A.L., Onuma Y. (2020). The usefulness of cardiac CT integrated with FFRCT for planning myocardial revascularization in complex coronary artery disease: A lesson from SYNTAX studies. Cardiovasc. Diagn. Ther..

[15] Nagumo S., Collet C., Nørgaard B.L., Otake H., Ko B., Koo B.-K., Leipsic J., Andreini D., Heggermont W., Jensen J.M. (2021). Rationale and design of the precise percutaneous coronary intervention plan (P3) study. Clin. Cardiol..

[16] Sonck J., Nagumo S., Nørgaard B.L., Otake H., Ko B., Zhang J., Mizukami T., Maeng M., Andreini D., Takahashi Y. (2022). Clinical validation of a virtual planner for coronary interventions based on coronary CT angiography. JACC Cardiovasc. Imaging.

[17] Belmonte M., Maeng M., Collet C., Nørgaard B.L., Otake H., Ko B., Koo B.-K., Mizukami T., Updegrove A., Barbato E. (2023). Accuracy of a virtual PCI planner based on coronary CT angiography in calcific lesions. J. Cardiovasc. Comput. Tomogr..

[18] Louvard Y., Medina A. (2015). Definitions and classifications of bifurcation lesions and treatment. EuroIntervention.

[19] Grodecki K., Opolski M.P., Staruch A.D., Michalowska A.M., Kepka C., Wolny R., Pregowski J., Kruk M., Debski M., Debski A. (2020). Comparison of computed tomography angiography versus invasive angiography to assess Medina classification in coronary bifurcations. Am. J. Cardiol..

[20] Kočka V., Thériault-Lauzier P., Xiong T.-Y., Ben-Shoshan J., Petr R., Laboš M., Buithieu J., Mousavi N., Pilgrim T., Praz F. (2020). Optimal fluoroscopic projections of coronary ostia and bifurcations defined by computed tomographic coronary angiography. JACC Cardiovasc. Interv..

[21] Michalowska A.M., Grodecki K., Staruch A.D., Kepka C., Wolny R., Pregowski J., Kruk M., Debski M., Debski A., Michalowska I. (2021). Visually estimated RESOLVE score based on coronary computed tomography to predict side branch occlusion in percutaneous bifurcation intervention. J. Thorac. Imaging.

[22] Lee S.-H., Lee J.M., Song Y.B., Park T.K., Yang J.H., Hahn J.-Y., Choi S.-H., Gwon H.-C., Lee S.-H., Kim S.M. (2019). Prediction of side branch occlusions in percutaneous coronary interventions by coronary computed tomography: The CT bifurcation score as a novel tool for predicting intraprocedural side branch occlusion. EuroIntervention.

[23] Grodecki K., Cadet S., Staruch A.D., Michalowska A.M., Kepka C., Wolny R., Pregowski J., Kruk M., Debski M., Debski A. (2020). Noncalcified plaque burden quantified from coronary computed tomography angiography improves prediction of side branch occlusion after main vessel stenting in bifurcation lesions: Results from the CT-PRECISION registry. Clin. Res. Cardiol..

[24] Pesarini G., Hellig F., Seth A., Shlofmitz R.A., Ribichini F.L. (2025). Percutaneous coronary intervention for calcified and resistant lesions. EuroIntervention.

[25] Sekimoto T., Akutsu Y., Hamazaki Y., Sakai K., Kosaki R., Yokota H., Tsujita H., Tsukamoto S., Kaneko K., Sakurai M. (2016). Regional calcified plaque score evaluated by multidetector computed tomography for predicting the addition of rotational atherectomy during percutaneous coronary intervention. J. Cardiovasc. Comput. Tomogr..

[26] Fujino A., Mintz G.S., Matsumura M., Lee T., Kim S.-Y., Hoshino M., Usui E., Yonetsu T., Haag E.S., Shlofmitz R.A. (2018). A new optical coherence tomography-based calcium scoring system to predict stent underexpansion. EuroIntervention.

[27] Zhang M., Matsumura M., Usui E., Noguchi M., Fujimura T., Fall K.N., Zhang Z., Nazif T.M., Parikh S.A., Rabbani L.E. (2021). Intravascular ultrasound-derived calcium score to predict stent expansion in severely calcified lesions. Circ. Cardiovasc. Interv..

[28] Conte E., Mushtaq S., Pontone G., Li Piani L., Ravagnani P., Galli S., Collet C., Sonck J., Di Odoardo L., Guglielmo M. (2020). Plaque quantification by coronary computed tomography angiography using intravascular ultrasound as a reference standard: A comparison between standard and last generation computed tomography scanners. Eur. Heart J. Cardiovasc. Imaging.

[29] Monizzi G., Sonck J., Nagumo S., Buytaert D., Van Hoe L., Grancini L., Bartorelli A.L., Vanhoenacker P., Simons P., Bladt O. (2020). Quantification of calcium burden by coronary CT angiography compared to optical coherence tomography. Int. J. Cardiovasc. Imaging.

[30] Okutsu M., Mitomo S., Onishi H., Nakajima A., Yabushita H., Matsuoka S., Kawamoto H., Watanabe Y., Tanaka K., Naganuma T. (2023). The estimation of coronary artery calcium thickness by computed tomography angiography based on optical coherence tomography measurements. Heart Vessels.

[31] Kurogi K., Ishii M., Ikebe S., Kaichi R., Takae M., Mori T., Komaki S., Yamamoto N., Tsujita K. (2023). Calcium evaluation using coronary computed tomography in combination with optical coherence tomography. Int. J. Cardiovasc. Imaging.

[32] Werner G.S., Hildick-Smith D., Martin-Yuste V., Boudou N., Sianos G., Gelev V., Rumoroso J.R., Erglis A., Christiansen E.H., Escaned J. (2023). Three-year outcomes of a randomized multicentre trial comparing revascularization and optimal medical therapy for chronic total coronary occlusions (EuroCTO). EuroIntervention.

[33] Obedinskiy A.A., Kretov E.I., Boukhris M., Kurbatov V.P., Osiev A.G., Ibn Elhadj Z., Obedinskaya N.R., Kasbaoui S., Grazhdankin I.O., Prokhorikhin A.A. (2018). The IMPACTOR-CTO trial. JACC Cardiovasc. Interv..

[34] Juricic S.A., Stojkovic S.M., Galassi A.R., Stankovic G.R., Orlic D.N., Vukcevic V.D., Milasinovic D.G., Aleksandric S.B., Tomasevic M.V., Dobric M.R. (2023). Long-term follow-up of patients with chronic total coronary artery occlusion previously randomized to treatment with optimal drug therapy or percutaneous revascularization of chronic total occlusion (COMET-CTO). Front. Cardiovasc. Med..

[35] Zhou H., Fan X., Yuan M., Wang W., Wu Q. (2024). Role of pre-procedure CCTA in predicting failed percutaneous coronary intervention for chronic total occlusions. Eur. J. Radiol. Open.

[36] Liang S., Bai Y., Zhang J., Wang A., Li J., Diao K., He Y. (2024). The added value of coronary CTA in chronic total occlusion percutaneous coronary intervention: A systematic review and meta-analysis. Eur. Radiol..

[37] Fujino A., Otsuji S., Hasegawa K., Arita T., Takiuchi S., Fujii K., Yabuki M., Ibuki M., Nagayama S., Ishibuchi K. (2018). Accuracy of J-CTO score derived from computed tomography versus angiography to predict successful percutaneous coronary intervention. JACC Cardiovasc. Imaging.

[38] Opolski M.P., Achenbach S., Schuhbäck A., Rolf A., Möllmann H., Nef H., Rixe J., Renker M., Witkowski A., Kepka C. (2015). Coronary computed tomographic prediction rule for time-efficient guidewire crossing through chronic total occlusion: Insights from the CT-RECTOR multicenter registry. JACC Cardiovasc. Imaging.

[39] Yu C.-W., Lee H.-J., Suh J., Lee N.-H., Park S.-M., Park T.K., Yang J.H., Song Y.B., Hahn J.-Y., Choi S.-H. (2017). Coronary computed tomography angiography predicts guidewire crossing and success of percutaneous intervention for chronic total occlusion: Korean multicenter CTO CT registry score as a tool for assessing difficulty in chronic total occlusion percutaneous coronary intervention. Circ. Cardiovasc. Imaging.

[40] Wang R., He Y., Xing H., Zhang D., Tian J., Le Y., Zhang L., Chen H., Song X., Wang Z. (2022). Inclusion of quantitative high-density plaque in coronary computed tomographic score system to predict the time of guidewire crossing chronic total occlusion. Eur. Radiol..

[41] Yu Y.-T., Sha Z.-Y., Chang S.-M., Zhai D.-T., Zhang X.-J., Hou A.-J., Feng W.-J., Li D.-W., Wang Y., Luan B. (2022). Accuracy of the Euro CTO (CASTLE) score obtained on coronary computed tomography angiography for predicting 30-minute wire crossing in chronic total occlusions. BMC Cardiovasc. Disord..

[42] Li J., Wang R., Tesche C., Schoepf U.J., Pannell J.T., He Y., Huang R., Chen Y., Li J., Song X. (2021). CT angiography-derived RECHARGE score predicts successful percutaneous coronary intervention in patients with chronic total occlusion. Korean J. Radiol..

[43] Maeremans J., Spratt J.C., Knaapen P., Walsh S., Agostoni P., Wilson W., Avran A., Faurie B., Bressollette E., Kayaert P. (2018). Towards a contemporary, comprehensive scoring system for determining technical outcomes of hybrid percutaneous chronic total occlusion treatment: The RECHARGE score. Catheter. Cardiovasc. Interv..

[44] La Scala E., Peyre J.-P., Maupas E., ReSurg, CT-CTO PCI Study Group (2023). Effect of preoperative coronary CT for planning of percutaneous coronary intervention for complex chronic total occlusion (CTS-C-CTOPCI): Study protocol for an open-label randomised controlled trial. Trials.

[45] Hong S.-J., Kim B.-K., Cho I., Kim H.-Y., Rha S.-W., Lee S.-H., Park S.M., Kim Y.H., Chang H.-J., Ahn C.-M. (2021). CT-CTO Investigators. Effect of coronary CTA on chronic total occlusion percutaneous coronary intervention: A randomized trial. JACC Cardiovasc. Imaging.

[46] Gondi K.T., Goyal A., Kane J., Allana S.S. (2024). Preprocedural planning for chronic total occlusion percutaneous coronary intervention. Am. J. Cardiol..

[47] Xenogiannis I., Jaffer F.A., Shah A.R., Omer M., Megaly M., Vemmou E., Nikolakopoulos I., Rangan B., Letter S. (2021). Computed tomography angiography co-registration with real-time fluoroscopy in percutaneous coronary intervention for chronic total occlusions. EuroIntervention.

[48] Opolski M.P., Knaapen P., Lesser J.R., Cavalcante J.L., Burke M.N., Brilakis E.S. (2021). Letter: How should coronary computed tomography angiography co-registration be applied in the chronic total occlusion hybrid algorithm?. EuroIntervention.

[49] Dai T., Wang J.-R., Hu P.-F. (2018). Diagnostic performance of computed tomography angiography in the detection of coronary artery in-stent restenosis: Evidence from an updated meta-analysis. Eur. Radiol..

[50] Decker J.A., O’Doherty J., Schoepf U.J., Todoran T.M., Aquino G.J., Brandt V., Baruah D., Fink N., Zsarnoczay E., Flohr T. (2022). Stent imaging on a clinical dual-source photon-counting detector CT system: Impact of luminal attenuation and sharp kernels on lumen visibility. Eur. Radiol..

[51] Hagar M.T., Soschynski M., Saffar R., Molina-Fuentes M.F., Weiss J., Rau A., Schuppert C., Ruile P., Faby S., Schibilsky D. (2024). Ultra-high-resolution photon-counting detector CT in evaluating coronary stent patency: A comparison to invasive coronary angiography. Eur. Radiol..

[52] Andreini D., Pontone G., Mushtaq S., Pepi M., Bartorelli A.L. (2010). Multidetector computed tomography coronary angiography for the assessment of coronary in-stent restenosis. Am. J. Cardiol..

[53] Masson R., Nkomo V.T., Holmes D.R., Pislaru S.V., Arsanjani R., Chao C.-J., Klanderman M., Abraham B., Morsy M., Fortuin F.D. (2024). Disproportionately high aortic valve calcium scores in atrial fibrillation: Implications for transcatheter aortic valve replacement. Eur. Heart J. Cardiovasc. Imaging.

